# Dorsal-approach open reduction for irreducible dislocation of the hallux interphalangeal joint: A case series

**DOI:** 10.1016/j.ijscr.2018.10.068

**Published:** 2018-11-01

**Authors:** Kanta Imao, Hitoshi Miwa, Kazutoshi Watanabe, Norio Imai, Naoto Endo

**Affiliations:** a Division of Orthopedic Surgery, Department of Regenerative and Transplant Medicine, Niigata University Graduate School of Medical and Dental Sciences, Asahimachi-dori 1, Niigata 951-8510, Japan; b Department of Orthopedic Surgery, Niigata Prefectural Shibata Hospital, Niigata 957-8588, Japan

**Keywords:** Interphalangeal joint, Irreducible dislocation, Sesamoid bone interposition, Plantar plate interposition

## Abstract

**Introduction:**

The common treatment for hallux interphalangeal joint dislocation is closed reduction, but some cannot be reduced by interposition of the sesamoid or the plantar plate in the interphalangeal joint, and such cases need open reduction.

**Presentation of case:**

In case 1, the patient was a 17-year-old boy who was injured when a motorcycle fell on his right hallux. X-ray revealed Miki type 2 IP joint dislocation. Because closed reduction failed, open reduction via the dorsal approach to the hallux IP joint was performed. In case 2, the patient was a 17-year-old boy who was hit by a car whilst walking. X-ray revealed Miki type 2 dorsal dislocation of the hallux IP joint. After closed reduction, X-ray and CT revealed that the IP joint dislocation changed from Miki type 2 to type 1. Open reduction was performed through the dorsal approach.

**Discussion:**

Fifteen cases of Miki type 2 changed to type 1 after closed reduction, and type 1 dislocation is often overlooked, leading to some chronic cases. X-rays should be performed after repositioning, specifically in lateral and oblique views.

**Conclusion:**

We experienced two cases of irreducible dislocation of the hallux IP joint. One of our cases was reclassified from Miki type 2 to type 1, but we did not overlook the failed closed reduction, as we performed CT. Hence, we recommend additional lateral-view X-rays and/or CT imaging.

## Highlights

• Unlike type 2, type 1 hallux interphalangeal joint dislocation is easy to miss.
• Excision of the sesamoid bone is unnecessary.
• External fixation for 3 weeks or K-wire fixation for 4 weeks is sufficient.

## 1 Introduction

Closed reduction is the usual treatment for hallux interphalangeal (IP) joint dislocation [1,2]. However, closed reduction of hallux IP joint dislocation is difficult when accompanied by sesamoid bone and plantar plate interposition in the joint, which needs open reduction. Hallux IP joint dislocation is relatively rare. We report two cases of hallux IP joint dislocation with failed closed reduction.

This retrospective case series has been reported in line with the PROCESS criteria [3]. Informed consent was obtained from the patients, and this case series was approved by our institutional review board.

## 2 Presentation of cases

**Fig. 1:**
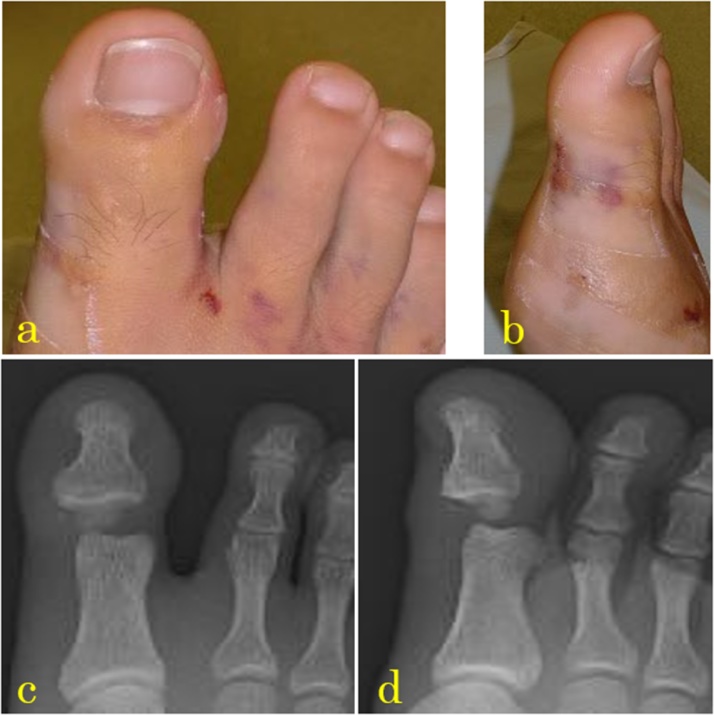
(a, b) After the failed closed reduction, the alignment was good, but the patient cannot move the joint voluntarily. (c, d) X-rays showing the sesamoid bone interposed in the IP joint.

**Fig. 2:**
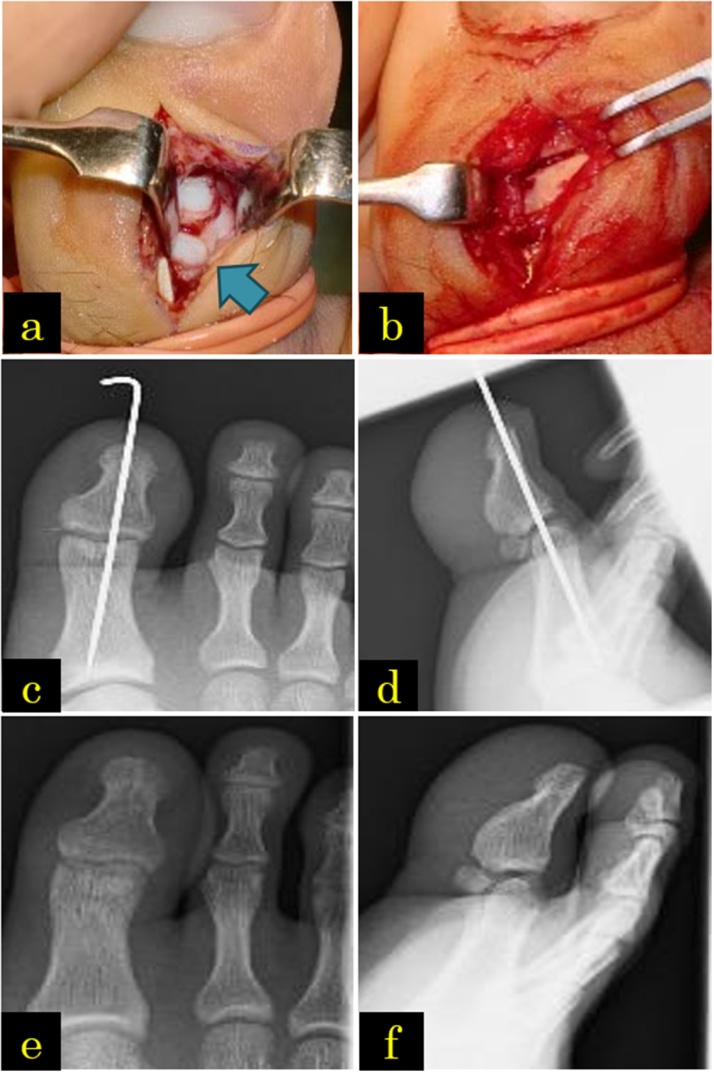
(a) Using the dorsal approach, the sesamoid bone can be seen interposed in the interphalangeal (IP) joint. (b) The sesamoid bone was pushed out with an elevator and reduced whilst pulling the distal phalanx. (c, d) After open repositioning, the unstable hallux IP joint underwent fixation using a 1.2-mm Kirshner wire. (e, f) During the last medical examination, no redislocation or osteoarthritis of the IP joint was noted.

**Fig. 3:**
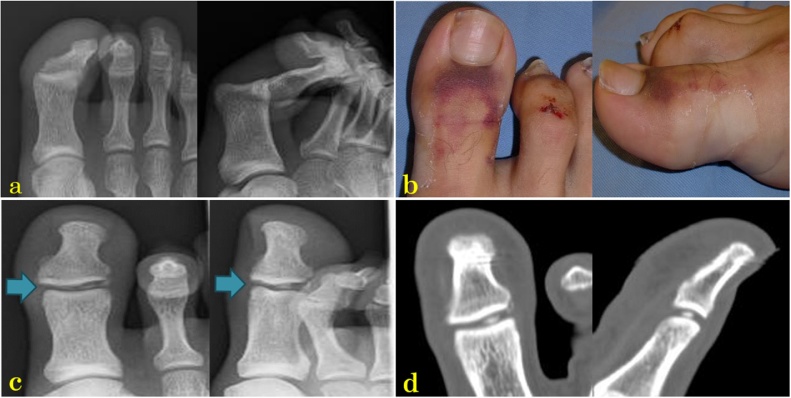
(a). X-rays during the first medical examination showing the posterior dislocation of the hallux interphalangeal (IP) joint. (b) After closed reduction, there was no macroscopic clear abnormality, but the joint cannot be moved. (c) The IP joint expanded a little, and a sesamoid spicule was noted in the IP joint. (d) Computed tomography showing the sesamoid spicule in the IP joint.

**Fig. 4:**
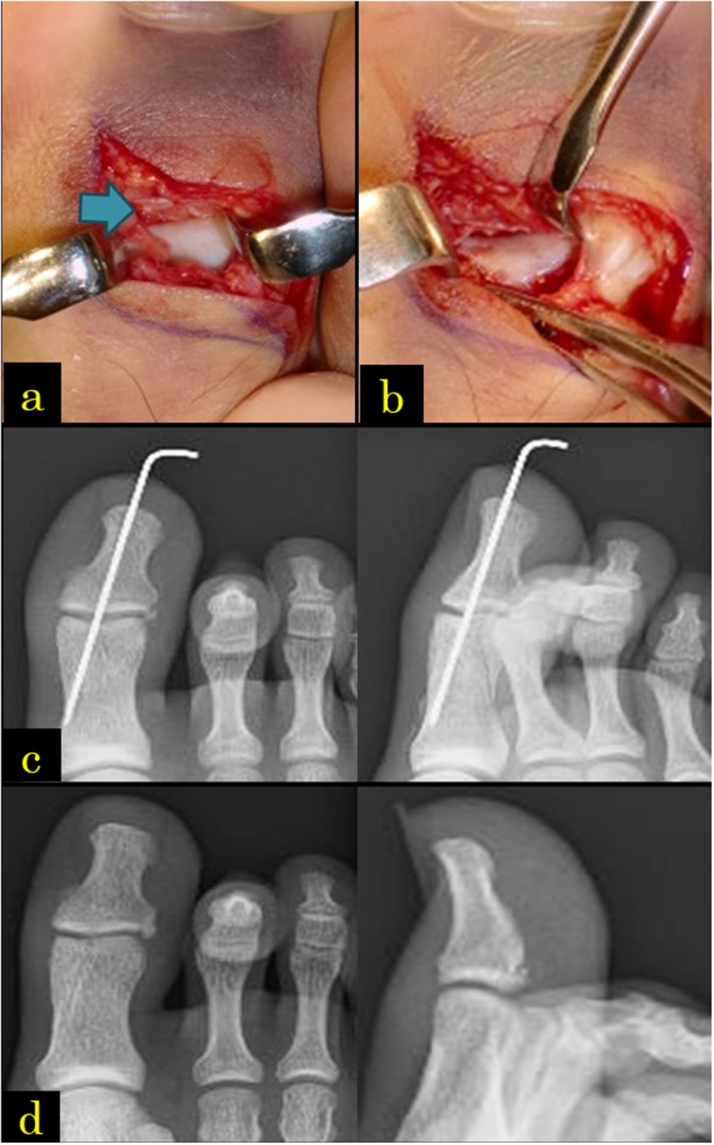
(a) Using the dorsal approach, a plantar plate was interposed in the hallux interphalangeal (IP) joint. (b) The plantar plate was pushed on the plantar side by an elevator and repositioned whilst the distal phalanx was being pulled. (c) Although IP joint had no apparent instability, it underwent fixation using a 1.2-mm Kirshner wire. (d) During the last medical examination, no expansion or osteoarthritis of the IP joint was noted.

In case 1, the patient was a 17-year-old boy who was injured when a motorcycle fell on his right hallux. He presented to our emergency department because of swelling and pain on his hallux. During the first medical examination, his hallux IP joint was fixed in hyperextension, restricting its movement (Fig. 1a, b). The nerve and vessels of the hallux were not affected. X-rays revealed that the sesamoid bone was interposed in the hallux IP joint, which was greatly expanded (Fig. 1c, d). Closed reduction of the dorsal dislocation of the IP joint was performed under digital block, with unsuccessful result; hence, open reduction was performed on the following day. Open reduction via the dorsal approach to the hallux IP joint was performed under digital block. We cut the lateral margin of the extensor hallucis longus tendon and confirmed that the sesamoid bone was interposed within the hallux IP joint (Fig. 2a). The sesamoid bone was pushed with an elevator to the plantar side and repositioned whilst traction was being applied to the distal phalanx. The extensor tendon was repaired, and the wound was closed. We fixed the IP joint with a 12-mm Kirshner wire (K-wire) (Fig. 2c, d). The postoperative course was unremarkable; for 3 weeks, he walked on his heel, with the ankle dorsiflexed and his toes not touching the ground. After 3 weeks, the K-wire was removed, and he walked with full weight bearing. After 2.5 months, he had no pain during rest or walking. The range of motion of the hallux IP joint was a little limited (injured toe: 0° extension, 10° flexion; opposite toe: 0° extension, 45° flexion) (Fig. 2e, f). In case 2, the patient was a 17-year-old boy who was hit by a car whilst walking, which led to hallux IP joint hyperextension. During the first medical examination in the emergency department, his hallux was swollen and hyperextended and he could not move the IP joint. The nerve and vessels were not damaged. X-ray revealed dorsal dislocation of the hallux IP joint (Fig. 3a). Closed reduction of the dorsal hallux dislocation was performed under digital block. His hallux had good alignment (Fig. 3b) but cannot be moved. It was fixed with a splint. X-ray revealed that the IP joint space was enlarged and a bony segment was interposed within the joint (Fig. 3c). Two days after the injury, we performed computed tomography (CT), which revealed that a sesamoid fragment was entrapped in the hallux IP joint (Fig. 3d). We performed open reduction using the dorsal approach under digital block. We cut the medial margin of the extensor hallucis longus tendon and confirmed the volar plate entrapped in the hallux IP joint (Fig. 4a). We pushed out the entrapped volar plate and sesamoid segment using an elevator to the plantar side and reduced the dislocation (Fig. 4b). We repaired the extensor hallucis longus tendon. After repositioning, no instability was noted. We fixed the IP joint with K-wire (Fig. 4c). After the surgery, he was able to do heel walks. Three weeks after surgery, the K-wire was removed and he started to walk with full weight bearing. Three months after surgery, the hallux IP joint had full range of motion (injured toe: 0° extension, 25° flexion; opposite toe: 10° extension, 15° flexion), and the patient can walk well without pain (Fig. 4d).

## 3 Discussion

Dislocations in the hallux usually occur in the metatarsophalangeal joint, and IP dislocations are rare, which is due to its strong fixation by soft tissues such as the extensor pollicis longus and flexor pollicis longus tendons, the volar plate and the collateral ligament [2,4]. Our cases had intrusion of the sesamoid bone and the volar plate, rendering repositioning impossible.

Miki et al. [5] classified closed reduction into two types: type 1, the sesamoid bone or the volar plate is interposed in the hallux IP joint; type 2, the sesamoid bone and the volar plate are dislocated dorsally on the head of the proximal phalanx of the hallux. There were 15 and 42 reported cases of types 1 [2,[5], [6], [7], [8]] and 2 [5,6,9], respectively; 5 cases were not classified [10].

Fifteen cases of Miki type 2 were reclassified as type 1 after closed reduction, and type 1 dislocation is often overlooked, leading to some chronic cases. Ward et al. [11] reported that X-rays should be done after repositioning to ensure hallux IP joint alignment. Additionally, Razak et al. [12] recommended that lateral-view X-ray should be performed, in addition to the anteroposterior and oblique views, to ensure that no Miki type 1 dislocation is overlooked. In case 2, we experienced a change in the Miki classification type 2 to type 1 after the reduction of dislocation, but the type 1 dislocation was not overlooked, as X-ray was done after repositioning, which revealed the expansion of the hallux IP joint. Furthermore, we also performed CT scan. Therefore, we believe that X-ray and CT scan should be performed after repositioning to avoid overlooking type 1 dislocation.

Most reports recommend closed reduction as the first choice of treatment of dorsal dislocation of the hallux IP joint. Yang et al. [13] reported that the first step in closed reduction was to dislocate the hallux dorsally to obtain hyperdorsiflexion. Next, the hallux was flexed to the plantar side with longitudinal traction. Then, a click must be felt and heard. Exact closed reduction can possibly decrease soft tissue damage, making close reduction more effective; further, the authors recommended that skill in closed reduction should be developed. However, Razak et al. [12] reported that open reduction should be done during the subacute and chronic phases, in contrast to the usual recommendation of performing it during the acute phase, which is based on the belief that delayed reduction is difficult due to scarring [14]. Some recommended open reduction if closed reduction fails, if the cause of persistent complication is unclear and if the X-ray does not show a widened IP joint [8,9,15]. In our cases, we immediately performed closed reduction of the dislocated hallux IP joint, but the result was unsuccessful; hence, we performed open reduction.

Open reduction can be performed using different approaches: plantar [6], plantar medial [16], lateral [10], medial [9] and dorsal [1,4,5,8,12,17]. The lateral approach has been performed in 20 cases [6,9] and is often used in the event of head fracture in the proximal phalanx or ligament rupture. The dorsal approach has been performed in 13 cases [2,8,12]. It is easy to perform and can be used to confirm and reposition the interposed tissue [4]. The plantar approach has been performed in five cases [6], and it is used to repair the volar plate. Recently, K-wire was inserted into the IP joint from the dorsal side percutaneously and the interposed tissues of the sesamoid bone and the volar plate were pushed to the plantar side 12,15].

In our cases, we performed the reduction using the dorsal approach and penetrated from the lateral margin of the extensor hallucis longus tendon. We could see the sesamoid bone and the volar plate that intervened, making the repositioning easy.

In many reports, the sesamoid bone was removed [2,6,7,10,12], but cases with preserved sesamoid bone had good results similar to those with removed sesamoid bone [4,5]. In both of our cases, the sesamoid bone was preserved, and both had good result. We believe that it is unnecessary to remove the sesamoid bone surgically if it can be repositioned successfully.

Yasuda et al. [6] reported that the repair of the volar plate is necessary to prevent hyperextension of the hallux IP joint and avoid redislocation [1]. However, some reports [4] stated that the restoration of the volar plate is unnecessary for improved prognosis, and in most reports, the volar plate was not repaired, leaving the stabilisation of the IP joint to the sesamoid bone [2]. In both of our cases, the volar plate was repositioned and not repaired; thus, the procedure used the dorsal approach.

## 4 Conclusion

We experienced two cases of irreducible dislocation of the hallux IP joint. One of our cases was reclassified from type 2 to type 1 in the Miki classification, but we did not overlook the failed closed reduction, as we performed CT. Unlike type 2, type 1 dislocation of the hallux IP joint is easy to miss. Hence, we recommend additional lateral-view X-rays and/or CT image. If the dislocation of the hallux IP joint is irreducible, dorsal-approach open reduction is the first choice for easy repositioning of the interposed soft tissue, sesamoid bone and volar plate. Based on reports and our experience, excision of the sesamoid bone and repair of the volar plate are unnecessary.

## Conflicts of interest

We declare that no grants or funds of benefits of any kind were received in support of the study.

## Sources of funding

We declare that no grants or funds of any kind were received in support of the study.

## Ethical approval

The patients provided informed consent, and the case report was approved by Niigata Prefectural Shibata Hospital’s ethics review board (approval number: 182).

## Consent

The patients provided informed consent, and the case report was approved by the appropriate ethics review board.

## Author contribution

KI: concept and planning of the case report, data interpretation and analysis, and drafting; KI, HM, KW, and NI: data collection; KI, HM, KW, NI, and NE: revision and approval of final manuscript; NE: design of study.

## Registration of research studies

The UIN of this study is 4133.

## Guarantor

Kanta Imao accepts full responsibility for the work and/or the conduct of the study, has access to the data, and controls the decision to publish.

## Patient perspective

The patients are satisfied because they are pain-free and back to their usual activities of daily living.

## Provenance and peer review

Not commissioned, externally peer reviewed.
